# Subunits of the Pyruvate Dehydrogenase Cluster of *Mycoplasma pneumoniae* Are Surface-Displayed Proteins that Bind and Activate Human Plasminogen

**DOI:** 10.1371/journal.pone.0126600

**Published:** 2015-05-15

**Authors:** Anne Gründel, Kathleen Friedrich, Melanie Pfeiffer, Enno Jacobs, Roger Dumke

**Affiliations:** TU Dresden, Institute of Medical Microbiology and Hygiene, Dresden, Germany; Miami University, UNITED STATES

## Abstract

The dual role of glycolytic enzymes in cytosol-located metabolic processes and in cell surface-mediated functions with an influence on virulence is described for various micro-organisms. Cell wall-less bacteria of the class *Mollicutes* including the common human pathogen *Mycoplasma pneumoniae* possess a reduced genome limiting the repertoire of virulence factors and metabolic pathways. After the initial contact of bacteria with cells of the respiratory epithelium via a specialized complex of adhesins and release of cell-damaging factors, surface-displayed glycolytic enzymes may facilitate the further interaction between host and microbe. In this study, we described detection of the four subunits of pyruvate dehydrogenase complex (PDHA-D) among the cytosolic and membrane-associated proteins of *M*. *pneumoniae*. Subunits of PDH were cloned, expressed and purified to produce specific polyclonal guinea pig antisera. Using colony blotting, fractionation of total proteins and immunofluorescence experiments, the surface localization of PDHA-C was demonstrated. All recombinant PDH subunits are able to bind to HeLa cells and human plasminogen. These interactions can be specifically blocked by the corresponding polyclonal antisera. In addition, an influence of ionic interactions on PDHC-binding to plasminogen as well as of lysine residues on the association of PDHA-D with plasminogen was confirmed. The PDHB subunit was shown to activate plasminogen and the PDHB-plasminogen complex induces degradation of human fibrinogen. Hence, our data indicate that the surface-associated PDH subunits might play a role in the pathogenesis of *M*. *pneumoniae* infections by interaction with human plasminogen.

## Introduction

The cell wall-less micro-organism *Mycoplasma pneumoniae* is an agent of atypical pneumonia in humans. Infections are most common in children and young adults, but all age groups can be affected. Up to nearly 30% of cases of community-acquired pneumonia are caused by this bacterium [1, 2]. Small-scale outbreaks have been mainly described in populations with close contact such as schools, colleges and army camps. In contrast, nation-wide or even world-wide peaks in the incidence of infections due to *M*. *pneumoniae* are reported every 3 to 7 years [1, 3].

With a genome size of 816 kbp and 688 open reading frames *M*. *pneumoniae* is one of the smallest self-replicating organisms known [4]. The limited genomic resources reduce the repertoire of metabolic pathways as well as of virulence factors. In consequence, these bacteria depend greatly on the host for successful *in vivo* replication. As a first step in colonization, the mycoplasmas adhere to the human respiratory mucosa, which is mediated by the attachment organelle, a unusual tip structure of the *M*. *pneumoniae* cell comprising a complex of adhesins and adherence-associated proteins [5]. The further infection process is associated with the release of hydrogen peroxide, superoxide anions [6–7] and the pertussis-like CARDS toxin [8] which results in interference with the integrity of the respiratory epithelium.

Because of the reduced genome, *M*. *pneumoniae* lacks the citric acid cycle and glycolysis is the major pathway to produce adenosine triphosphate (ATP). Despite the fact that this process takes place in the cytosol of the cells, glycolytic enzymes can be transported by an unknown mechanism to the surface of the microorganisms [9], where they may interact with host factors. First reported for streptococci, this phenomenon seems a common feature as it is described in phylogenetically different organisms including gram-negative and -positive bacteria, fungi and parasites [10–12]. Glyceraldehyde-3-phosphate dehydrogenase (GAPDH) and enolase of various organisms are typical examples for surface-displayed glycolytic enzymes that interact with proteins of the human extracellular matrix (ECM). The number of host ECM components which are described as binding partners of microbial glycolytic enzymes has increased in recent years [12–14]. Since these interactions are known in different pathogenic microorganisms, a general role in host-microbe interaction and also as virulence factor cannot be excluded.

Surface localization of glycolytic enzymes and their interaction with host components were also described for different mycoplasma species [15–19]. In *M*. *pneumoniae*, the E1β subunit of pyruvate dehydrogenase (PDHB) is able to interact with human fibronectin [20] and with human plasminogen [21]. Furthermore, GAPDH was characterized as binding partner of human fibrinogen [22]. However, analysis of the Triton X (TX)-100 insoluble proteins of total cells indicates that further glycolytic enzymes can be found in the fraction of membrane-associated proteins of *M*. *pneumoniae* [23] including subunit A of the PDH complex. In addition, this subunit is known to be a protein which is complexed to the P1 protein [24], the major adhesin of *M*. *pneumoniae*. These aspects suggest that, besides PDHB, further PDH subunits can be cell surface-exposed and, in consequence, can function as possible interaction partners of human ECM proteins. The PDH complex consists of four enzymatic components: pyruvate dehydrogenase major (E1α; PDHA) and minor (E1β) subunits, dihydrolipoyl acetyltransferase (E2; PDHC) and dihydrolipoyl dehydrogenase (E3; PDHD). The components are encoded by the genes *pdhA* (*mpn393*), *pdhB* (*mpn392*), *pdhC* (*mpn391*) and *pdhD* (*mpn390*) [4]. These are organized in two operons encoding for the E1α and E1β subunits and the other for the E2 and E3 subunits ([25]; http://csbl.bmb.uga.edu/DOOR/, http://operons.ibt.unam.mx/OperonPredictor/).

In the present study, subunits of the PDH complex of *M*. *pneumoniae* were characterized as cluster of plasminogen-binding proteins which are displayed on the surface of the cell. Furthermore, PDHB was able to activate plasminogen and the complex degrades human fibrinogen. The data contribute to the understanding of the infection process due to an important human pathogen with a minimal genome.

## Material and Methods

### Bacteria, cell line and growth conditions

The *M*. *pneumoniae* strain M129 (ATCC 29342), the *Escherichia coli* strains NovaBlue (Novagen, Darmstadt, Germany, #70181–3) and BL21(DE3) (Novagen, #70235), and the HeLa cells (human cervical carcinoma cell line, ATCC CCL-2) were grown as described [21, 22]. Protein concentration of cells was measured with the BCA protein assay kit (Pierce, Rockford, IL, USA, #23227) as recommended by the manufacturer.

### Expression and purification of recombinant proteins

DNA was isolated from *M*. *pneumoniae* using the QIAamp DNA mini kit (Qiagen, Limburg, Netherlands, #51304) as described by the manufacturer (blood and fluid protocol). PCR with primers (Table 1) amplified the genome region surrounding the genes coding for PDHA-D. The products of the PCR were purified with the QIAquick PCR purification kit (Qiagen, #28104) and served as target for the multiple mutation reaction (MMR; [26]) to change the TGA codons to TGG during the amplification of the complete genes. Cloning into the pET-30/LIC vector (Novagen, #69077–3), transformation of *E*. *coli* strains NovaBlue and BL21(DE3), sequencing of inserts, expression, purification and concentration of the N-terminal 6x His-tagged recombinant proteins were performed as reported recently [21]. Protein concentration was measured as described.

**Table 1:** Oligonucleotides used in the study (underlined: vector-specific sequence; double underlined: mutation of TGA to TGG).

Target	Oligonucleotide	Sequence (5‘- 3‘)	Description
*PDHA (mpn393)*	MpPDHAf1	GTT AGC AGC CGT GTT GCA AG	Amplification and sequencing
	MpPDHAf2	CAA CGA ACA GTT AAA GCA CGC	
	MpPDHAf3	CAA AAC GCT GCC CAT TAA C	
	MpPDHAf4	GCG AGT TAC GAA GCA ATG C	
	MpPDHAr	GAT AGA TCC ATT GCG TTA CCC	
*PDHB (mpn392)*	MpPDHBf1	GTT GAC TCC TGA TCT AGC GC	
	MpPDHBf2	CAT GCC AAT GGG TGG TG	
	MpPDHBf3	GGG TAA CGC AAT GGA TCT AG	
	MpPDHBf4	GGA CAA GGG CAT TGA ACT C	
	MpPDHBr	CTA GGT GCA ATT ATT CTA ACA AAC	
*PDHC(mpn391)*	MpPDHCf	CAG CTA ACT GCA GTA AAT GCA AC	
	MpPDHCr	CAC ACG CCA CCA AAG TAT TC	
*PDHD(mpn390)*	MpPDHDf1	CAT GGA AGA ACG TGT TGT GC	
	MpPDHDf2	CAA GAT TGT CGA TTA CCT CC	
	MpPDHDr	CTT CTA ACG CTG CGT TGA AG	
*PDHA (mpn393)*	MpPDHAVf	GAC GAC GAC AAG ATG GCA ATT TTG ATT AAA AAC	MMR* and transformation
	MpPDHAM1	P- GCT TGT TTG GCA ACG TGC TG	
	MpPDHAM2	P- GCT TTT ACT TTA CTG GAA CGG TAA C	
	MpPDHAM3	P- CCA TTC ACA AGT GGA ACT CGG	
	MpPDHAM4	P- CTT CTC TTG GCG TCA AGG TC	
	MpPDHAVr	GAG GAG AAG CCC GGT TTA GTC TTT AAA GTA TTT TTT AGC	
*PDHB (mpn392)*	MpPDHBVf	GAC GAC GAC AAG ATG TCA AAA ACA ATT CAA GCA AAT AAC	
	MpPDHBM	P-CGT ACC ATT TCC CCT TGG GAC AAG	
	MpPDHBVr	GAG GAG AAG CCC GGT TTA CTT TAA AAG TTG GTT AAC AGC TTC	
*PDHC(mpn391)*	MpPDHCVf	GAC GAC GAC AAG ATG GCA AAT GAG TTT AAG TTC AC	
	MpPDHCVr	GAG GAG AAG CCC GGT CTA AGC TAC TTC TAA ATC AAT TAA TTC	
*PDHD(mpn390)*	MpPDHDVf	GAC GAC GAC AAG ATG AAT TAC GAT CTC ATT ATT ATA G	
	MpPDHDM	P-GTA GCA CTT ACC TGG AAC CAA TTG	
	MpPDHDVr	GAG GAG AAG CCC GGT CTA TTT AAA GTG ATC AAA AAG AGC	

* Multiple Mutation Reaction [26]

### Production of polyclonal antisera

Anesthetized guinea pigs (Charles River, Sulzfeld, Germany) were used for immunization. Animal experiments were carried out in accordance with the recommendations of the Federation of Laboratory Animal Science Associations (FELASA) and approved by the ethical board of Landesdirektion Sachsen, Dresden, Germany (permit no. 24–9168.25-1/2011-1). Primary subcutaneous immunization of guinea pigs with total *M*. *pneumoniae* antigen and recombinant proteins rPDHA-D, booster immunizations and sera collection were done as reported [21].

### SDS-PAGE, Western Blotting and ELISA

The result of expression of recombinant proteins rPDHA-D was analyzed by SDS-PAGE using 10% NuPage Bis-Tris gels (Life, Darmstadt, Germany, #NPO303 Box) followed by Coomassie staining (Merck, # 115444). To confirm the specificity of the produced polyclonal antisera, blotted *M*. *pneumoniae* whole cells were incubated with the antisera to rPDHA-D (1:500) and detected with anti-guinea pig IgG (1:750; Dako, Hamburg, Germany, #P01402-2). Chemiluminescence signals were digitally recorded using a LAS-300 imager (Fujifilm, Düsseldorf, Germany).

To compare the quantitative reactivity of sera by ELISA, cavities of 96-well plates (Greiner, #655001) were coated overnight with *M*. *pneumoniae* total proteins (15 μg/ml) and blocked as described [21]. Wells were incubated with guinea pig sera to whole proteins of *M*. *pneumoniae* and to subunits of the PDH complex (1:250) followed by anti-guinea pig IgG (1:750). After incubation with the substrate (TMB super slow, Sigma, #T5569), the reaction was stopped with 1 M HCl and absorbance was detected at 450 nm with reference wavelength of 620 nm.

### Fractionation of *M*. *pneumoniae* total proteins

For the localization of proteins PDHA-D in the mycoplasma cell, the total proteins of freshly grown *M*. *pneumoniae* cells were separated into membrane and cytosol fractions by ultracentrifugation as described [21]. To control the performance of fractionation in ELISA experiments, plate wells were coated with the cytosolic and membrane fraction of *M*. *pneumoniae* total proteins (15 μg/ml). Detection was performed with guinea pig sera against the subunits of the PDH complex (1:250) followed by anti-guinea pig IgG (1:750). The serum to the conserved surface-exposed C-terminal part of the main P1 adhesin (rP12, [27]) served as control for a membrane-associated protein. Based on the extensive characterization of this protein [21], enolase was used as control for a cytosolic and not surface-exposed protein of *M*. *pneumoniae*.

### Surface localization of subunits of the PDH complex

Identification of cellular localization of PDHA-D is fundamental for further conclusions about the role of these proteins in pathogenesis and was studied with different experimental approaches. For fluorescence microscopy, freshly grown *M*. *pneumoniae* cells were inoculated in cavities of a chamber slide (Thermo, #154526) and treated as reported [21]. For parallel detection of surface structures of fixed mycoplasma cells, mixtures of guinea pig antibodies to the subunits of the PDH complex with rabbit antiserum to the TX-100 insoluble fraction of *M*. *pneumoniae* total proteins (1:250 each) were added to the cavities. Guinea pig pre-immune serum and guinea pig antiserum obtained after three subcutaneous immunizations with *M*. *pneumoniae* total proteins (1:250) in combination with a rabbit antiserum to the TX-100 insoluble fraction were used as negative and positive controls, respectively. Detection was carried out with a mixture of FITC-labeled anti-guinea pig IgG (Sigma, #F6261) and TRITC-labeled anti-rabbit IgG (Sigma, #T6778, 1:500 each). Immunofluorescence of cells was checked at 400x with a fluorescence microscope (Axioskop, Zeiss, Jena).

The colony blot technique was used to detect the occurrence of PDH subunits on the surface of the bacteria [21]. Pieces of nitrocellulose with blotted proteins were treated with guinea pig antisera (1:250 each) to rPDHA-D, to total proteins of *M*. *pneumoniae* (positive control), to *M*. *pneumoniae* enolase and a pre-immune serum (both as negative controls). Detection was carried out as described by using peroxidase-conjugated anti-guinea pig IgG (1:750).

To further assess surface-localized regions of the proteins PDHA-D, mild proteolysis of *M*. *pneumoniae* cells with trypsin (Sigma, #T1426) was carried out. Pre-examination of cleavage frequency of proteins analyzed (http://web.expasy.org/peptide_cutter/) resulted in numbers of cleavage sites of 13 (C-terminal part of P1 adhesin), 32 (PDHB), 37 (PDHC), 40 (PDHA), 44 (PDHD) and 46 (enolase), respectively. Trypsin treatment of cells was done as reported [21]. In parallel, recombinant proteins enolase and PDHD were treated to confirm digestibility. Blotted trypsin-treated and untreated proteins were incubated with the antisera to recombinant proteins rPDHA-D (1:250 each) and sera against enolase and the C-terminal part of P1 protein of *M*. *pneumoniae* as controls. Anti-guinea pig IgG (1:750) was used for detection.

### Binding of recombinant proteins to HeLa cells

Wells of ELISA plates were coated for 2 hours at 37°C with freshly harvested HeLa cells (100 μg/ml) and in parallel with rPDHA-D (10 μg/ml). The recombinant protein rP12 confirmed as involved in cytoadherence acted as positive control [27]. Recombinant protein rP8 (part of middle region of the P1 protein) which was characterized as not able to bind to HeLa cells was used as negative control [27]. Eluates obtained after processing lysed *E*. *coli* BL21(DE3) cells through Ni-NTA-agarose columns were included to test the influence of remaining *E*. *coli* proteins on binding to HeLa cells. After blocking, recombinant proteins rPDHA-D, rP8, rP12 and *E*. *coli*-specific proteins (10 μg/ml) were added to cell-coated wells and incubated for 2 hours at 37°C. Simultaneously, wells with immobilized proteins were incubated with PBS under the same experimental conditions. After washing, proteins in wells with and without HeLa cells were detected with guinea pig sera to the six recombinant proteins and to the *E*. *coli* proteins (1:1000) followed by peroxidase-labeled anti-guinea pig IgG (1:1000).

To test the influence of the antisera against the proteins of the PDH complex on the binding to HeLa cells, wells of ELISA plates were coated with freshly harvested HeLa cells for 2 hours at 37°C (100 μg/ml). In parallel, recombinant proteins rPDHA-D (50 μg/ml) were pre-incubated with the corresponding antisera (1:50 in PBS) for 1.5 hours at room temperature in an overhead shaker. Recombinant protein GAPDH [22] was also treated with sera against the recombinant proteins PDHA-D to analyze cross-reactions of the sera with a non-corresponding protein. Treated recombinant proteins rPDHA-D and rGAPDH were added to the human cells and incubated for 2 hours at 37°C. After washing, bound recombinant proteins were detected by incubating the wells with the corresponding antisera (1:1000) and anti-guinea pig IgG (1:1000).

### Adhesion inhibition assay

An ELISA-based assay was used to quantify the influence of the antisera to proteins of the PDH complex on the adhesion of *M*. *pneumoniae* to HeLa cells. ELISA plates were coated with freshly harvested HeLa cells (100 μg/ml) for 2 hours at 37°C. Simultaneously, freshly grown *M*. *pneumoniae* cells were prepared as described and resuspended in PBS to an OD_660_ of 0.44. The suspension was sheared through a 27-gauge needle (B. Braun, Melsungen, Germany, #9161627S) to reduce self-aggregation of bacteria. The mycoplasma suspension was diluted 1:20 in PBS containing 50% PPLO medium and 10% heat-inactivated antiserum to recombinant proteins PDHA-D, to total antigen of *M*. *pneumoniae* (positive control) or pre-immune guinea pig serum (negative control). The mixtures were incubated for 1.5 hours at 37°C with head-over-head rotation and added to the blocked wells of ELISA plates for 1.5 hours at 37°C with mild shaking. After washing, bound bacteria were detected by incubating the wells with the rabbit antiserum against the TX-100 insoluble fraction (1:500) and peroxidase-conjugated anti-rabbit IgG (Dako, #P039901-2, 1:1000). Three independent experiments with eight parallels each were carried out.

### Binding of human plasminogen

The interaction of human plasminogen with *M*. *pneumoniae* proteins was analyzed as described recently [21]. Briefly, after separation by SDS-PAGE, the recombinant proteins rPDHA-D, total proteins of *M*. *pneumoniae* and BSA (100 μg each) were blotted onto a nitrocellulose membrane. Human plasminogen (5 μg/ml, Sigma, #P7999) was added and incubated for 2 hours at room temperature on a shaker. PBS was used as control. Following three washing steps, the membranes were incubated with rabbit anti-plasminogen (1:3000, Sigma, #HPA021602) for 2 hours at room temperature. Bound plasminogen was detected with peroxidase-conjugated anti-rabbit IgG (1:1000).

Concentration-dependent binding of plasminogen to recombinant proteins was quantified by ELISA [21]. Wells were coated with recombinant proteins rPDHA-D (10 μg/ml). Different concentrations of plasminogen (0.1, 0.25, 0.5, 0.75, 1 and 5 μg/ml) and PBS were added for 2 hours at 37°C. Bound plasminogen was detected with anti-plasminogen (1:2000) and anti-rabbit IgG (1:1000).

In addition, the influence of the corresponding antisera to subunits of the PDH complex on binding of human plasminogen to *M*. *pneumoniae* cells and to the recombinant proteins rPDHA-D was tested. Wells of ELISA plates were coated with the recombinant proteins (15 μg/ml) or total proteins of *M*. *pneumoniae* (25 μg/well). Increasing concentrations of plasminogen (0.25, 0.5, 1.0, 5 μg/ml) in PBS were incubated with antiserum against PDHA-D and guinea pig pre-immune serum as control (1:100 each) for 1.5 hours at room temperature with overhead rotation. The mixtures were added to immobilized antigens and incubated for 2 hours at 37°C. Bound plasminogen was detected with anti-plasminogen (1:500) and peroxidase-conjugated anti-rabbit IgG (1:750) as described.

To analyze the role of ionic interactions in plasminogen binding [28], 96-well plates were coated overnight with recombinant proteins rPDHA-D (10 μg/ml) at 4°C. After washing and blocking, human plasminogen (10 μg/ml PBS) with increasing concentrations of NaCl (Roth, #9265.2) or heparin (Sigma, #H3393) were added to bound proteins and incubated for 2 hours at 37°C. Bovine serum albumin (BSA, Pierce, #23209) was used as negative control. Bound plasminogen was detected using rabbit anti-plasminogen (1:500) and anti-rabbit IgG (1:750) as described above.

To investigate the influence of lysine residues on plasminogen binding, the lysine analog ε- aminocaproic acid (1mM, Sigma, #07260, [28]) and human plasminogen (10 μg/ml PBS) were added to rPDHA-D or BSA (10 μg/ml each) and immobilized in wells of an ELISA plate. As control for a plasminogen-binding protein part that is insensitive to the lysine analog, peptide 3 of the panel of peptides overlapping PDHB was used [21]. Detection was carried out as described above.

### Plasminogen activation assay

The ability of subunits of PDH complex of *M*. *pneumoniae* to activate plasminogen to plasmin was monitored in a quantitative assay as described [29]. Briefly, wells of ELISA plates were coated with recombinant proteins rPDHA-D or BSA as negative control (10 μg/ml each) and blocked. Human plasminogen (10 μg/ml PBS) was added and incubated for 2 hours at 37°C. After washing, human uPA (4 ng per well; Millipore, #CC400) and the plasmin-specific substrate D-valyl-leucyl-lysine-p-nitroanilide dihydrochloride (0.4 mM; Sigma, #V0882) were added. The plates were incubated overnight at 37°C and plasmin was detected at 405 nm.

### Fibrinogen degradation assay

To analyze whether the proteins of the PDH complex are able to degrade fibrinogen, an assay was performed as reported [30]. Recombinant proteins rPDHA-D, total proteins of *M*. *pneumoniae* or BSA (10 μg/ml of each) were immobilized in wells of a 96-well plate as described. Human plasminogen (10 μg/ml PBS) was added for 2 hours at 37°C. After washing, human uPA (80 ng/ml) and human fibrinogen (20 μg/ml, plasminogen-depleted, Millipore, # 341578) was incubated at room temperature. After different time points, aliquots were taken and the reaction was stopped by heating for 10 minutes at 56°C. The heat-inactivated samples were used to coat 96-well plates overnight at 4°C. After washing and blocking, the degradation of fibrinogen was analyzed using goat anti-fibrinogen (1:1000, Sigma, #F8512) for 90 minutes at room temperature. Detection was carried out with peroxidase-conjugated anti-goat IgG (1:1000, Sigma, #A4174).

## Results

### Production of recombinant proteins rPDHA-D of *M*. *pneumoniae* and generation of monospecific antisera

**Fig 1:**
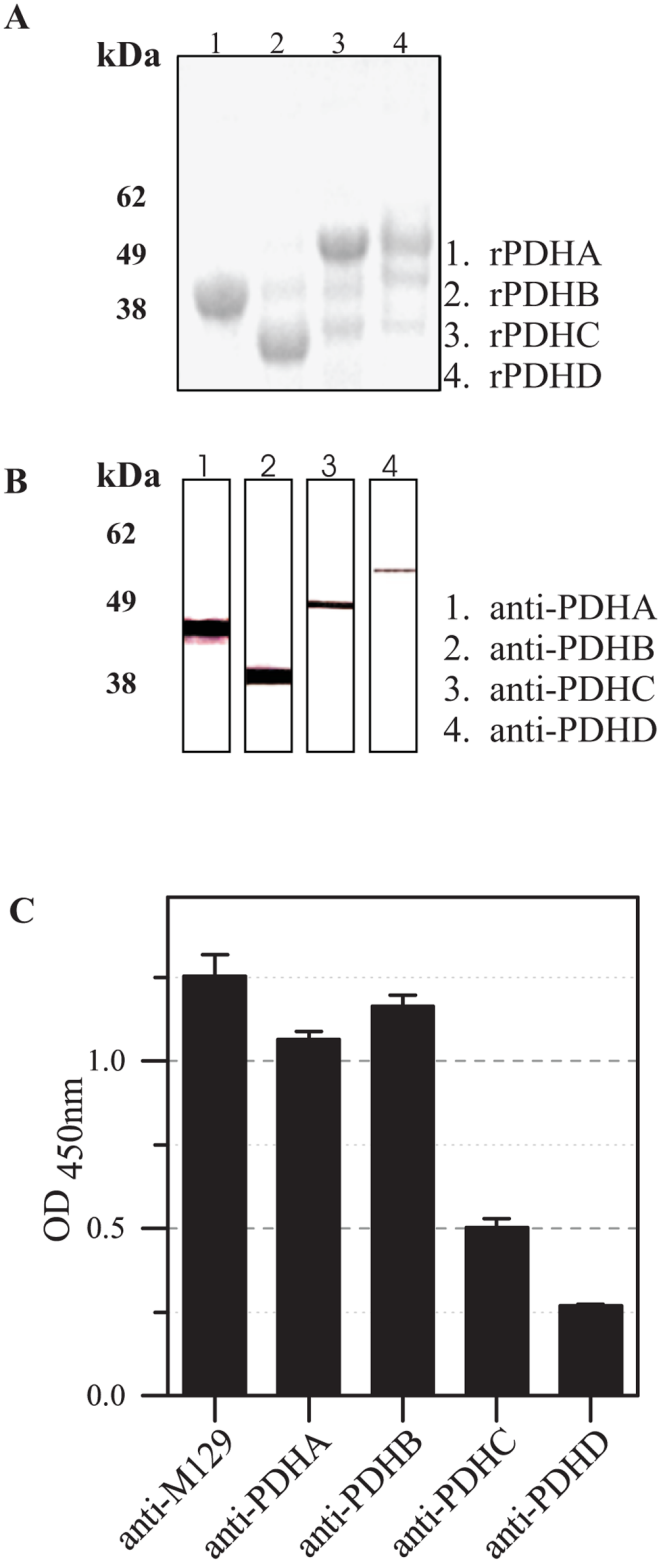
Production and reaction of recombinant proteins rPDHA-D and corresponding polyclonal guinea pig antisera. EK-LIC vector and *E*. *coli* BL21(DE3) were used to produce recombinant proteins. (A) Proteins were purified, concentrated, separated by SDS-PAGE and stained with Coomassie. (B) Western blot reaction of total proteins of *M*. *pneumoniae* M129 with antisera to rPDHA-D. (C) Results of ELISA experiments to analyze the reactivity of antisera to total proteins and to PDH subunits A-D with whole antigen of *M*. *pneumoniae* M129. Data represent means and standard deviations of eight parallels.

After exchanging the TGA codons in three of four genes, the complete genes encoding for subunits of the PDH complex were amplified and cloned into the pET-30 vector. The proteins were expressed in *E*. *coli*, purified by affinity chromatography and separated by SDS-PAGE. Coomassie staining of gels resulted in prominent products with the expected molecular weights (Fig 1A). In addition, blotted recombinant proteins were detected with anti-His antibodies confirming these prominent bands specifically (data not shown).

The recombinant proteins were used to produce polyclonal guinea pig antisera. The specificity was tested by incubation of sera to rPDHA-D with blotted total *M*. *pneumoniae* proteins. Exclusive signals were found at the predicted molecular weights of these proteins (Fig 1B). The quantitative reactivity of anti-rPDHA-D in comparison to an antiserum to total proteins of *M*. *pneumoniae* was analyzed using an ELISA with immobilized *M*. *pneumoniae* total proteins as antigens. Comparable OD values regarding the sera to rPDHA, rPDHB and total proteins of *M*. *pneumoniae* were found (Fig 1C). Sera to rPDHC and rPDHD showed significantly reduced reactivity indicating a low density of epitopes, a lower affinity of antibodies and/or a low concentration of both antigens among total *M*. *pneumoniae* proteins.

### Localization of subunits of the PDH complex in *M*. *pneumoniae* cells

**Fig 2:**
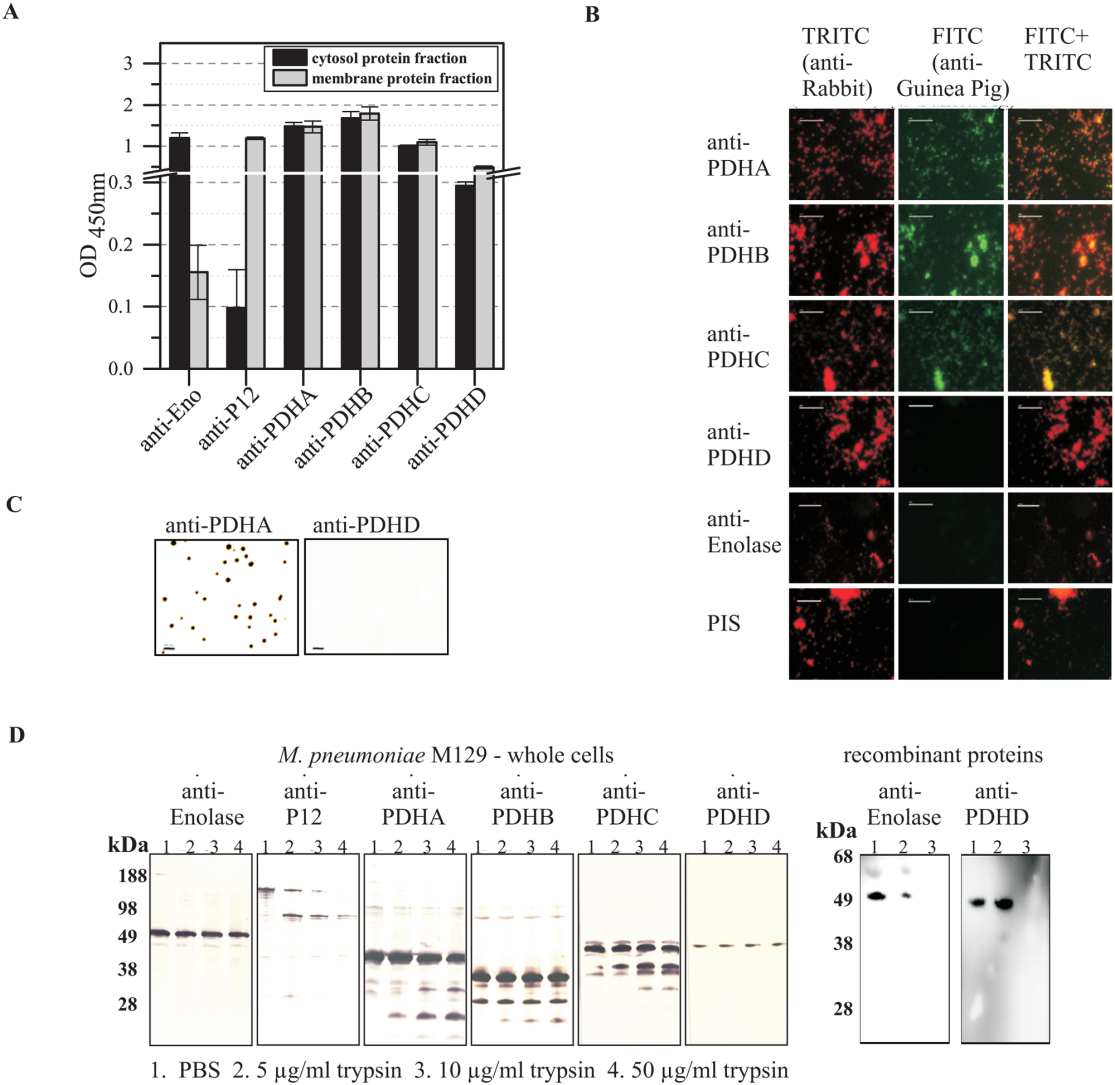
Localization of PDHA-D in *M*. *pneumoniae* cells. (A) Reactivity of guinea pig anti-rPDHA-D (1:250) with immobilized membrane and cytosolic proteins of *M*. *pneumoniae* M129. Sera to the surface-exposed C-terminal part of the main P1 adhesin (P12) and to cytosolic enolase (Eno) of *M*. *pneumoniae* acted as controls. Data show means and standard deviations of eight parallels. (B) Immunofluorescence of fixed *M*. *pneumoniae* cells treated with a mixture of guinea pig anti-rPDHA-D and rabbit anti-TX-100 insoluble protein fraction (positive control). Mixture of rabbit anti-TX-100 insoluble protein fraction and guinea pig anti-enolase as well as guinea pig pre-immune serum (PIS) act as negative controls. Detection was carried out by incubation with FITC-conjugated anti-guinea pig IgG and TRITC-conjugated anti-rabbit IgG. Bar: 10 μm. **(C)** Immunoblot reaction of 8 days-old *M*. *pneumoniae* M129 colonies. Colonies were covered with nitrocellulose membrane followed by incubation with sera to recombinant proteins PDHA-D. The reaction of anti-PDHA and anti-PDHD is illustrated as an example. Further positive signals were obtained after incubation of blots with anti-PDHB, anti- PDHC and the positive control anti-rP12, respectively (data not shown). The control anti-enolase demonstrated a negative result. Bar: 200 μm. (D) Results of the trypsin treatment of freshly grown *M*. *pneumoniae* M129 cells. Harvested bacteria were treated with increasing concentrations of trypsin or PBS as control. The reaction was stopped by boiling, samples were separated by SDS-PAGE and blotted. Nitrocellulose membranes were incubated with sera to cytosolic enolase (negative control), the surface-exposed near C-terminal part of adhesin P1 (P12; positive control) and to recombinant PDHA-D, respectively. Treatment of recombinant proteins enolase and PDHD with trypsin was used to confirm digestibility.

The ELISA reaction of the antibodies to recombinant proteins rPDHA-C with the membrane and cytosolic protein fraction of *M*. *pneumoniae* whole cells demonstrated the distribution of these glycolytic enzymes in both compartments (Fig 2A). The cytosolic enzyme enolase and the surface-exposed C-terminal part of the main P1 adhesin were used as controls for the appropriate performance of the test. The results are confirmed by ELISA experiments using the TX-100 soluble and TX-100 insoluble protein fractions [23] as well as the protein fractions obtained after using the ProteoExtrakt Transmembrane Protein Extraction Kit (Merck) as antigens (data not shown).

To prove the occurrence of glycolytic enzymes on the surface of mycoplasma cells, grown bacteria were fixed and incubated with guinea pig sera to recombinant proteins rPDHA-C of *M*. *pneumoniae* (Fig 2B). Surface-displayed proteins were detected by fluorescence microscopy using FITC- and TRITC-labeled secondary antibodies against guinea pig and rabbit IgG. Strong signals were obtained with sera against PDHA-C, whereas cells treated with sera to PDHD and to enolase remained negative. Incubation with guinea pig pre-immune serum confirmed that unspecific cross-reactions are not assumed.

In colony blot experiments using 8-day-old agar cultures, antisera to PDHA-C and to total proteins of *M*. *pneumoniae* reacted strongly, but no signals were obtained using the serum to PDHD (Fig 2C). The absence of reactivity of antiserum to enolase and of guinea pig pre-immune serum indicates that detection of cytosolic proteins and nonspecific reactions were excluded.

Incubation of trypsin-treated and blotted *M*. *pneumoniae* cells with antisera to recombinant PDH subunits yielded partial degradation products of proteins PDHA-C (Fig 2D). In contrast, PDHD was stable during these experimental conditions. The stability of the cytosolic glycolytic enzyme enolase (negative control) indicates integrity of the cell membrane after trypsin treatment whereas the C-terminal part of the P1 adhesin (positive control) was completely digested. Digestion of recombinant proteins enolase and PDHD confirmed cleavability of these proteins. Further experiments demonstrated that *M*. *pneumoniae* colonies treated with trypsin showed a decrease of PDHA-C-positive colonies of 30% in comparison to untreated colonies (data not shown).

Taking the results of localization experiments together, all four PDH subunits can be found in both fractions of membrane-associated and cytosolic proteins of *M*. *pneumoniae*. Furthermore, PDHA-C occur in high concentrations on the surface of mycoplasma cell whereas PDHD does not.

### Interaction of HeLa cells and human plasminogen with recombinant PDHA-D

**Fig 3:**
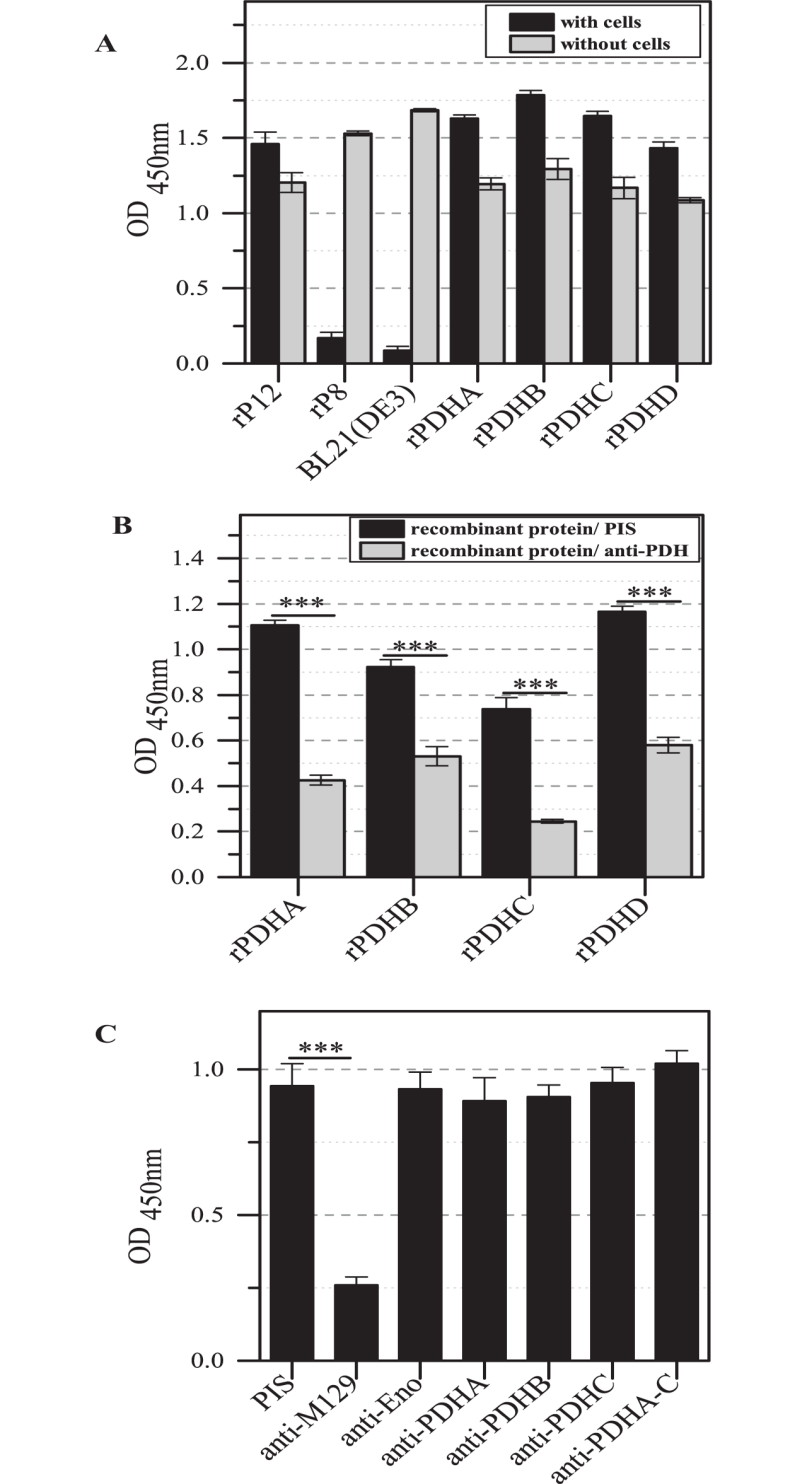
Binding of recombinant proteins rPDHA-D to human cells. (A) ELISA results after incubation of recombinant proteins with immobilized HeLa cells and detection with antisera to PDHA-D. OD values were compared with wells coated with recombinant proteins. Recombinant protein rP8 (middle part of the P1 protein without interaction to human cells) and remaining *E*. *coli*-specific proteins after Ni-agarose affinity chromatography (BL21(DE3)) acted as controls. The C-terminal part of the P1 protein (rP12) was used as positive control. Data represent means and standard deviations of eight parallels. (B) Influence of sera against recombinant proteins on binding of rPDHA-D to HeLa cells. rPDHA-D were pre-incubated with antisera to PDHA-D and added to immobilized HeLa cells. Incubation of recombinant proteins with guinea pig pre-immune serum was used as control. Data represent means and standard deviations of eight parallels (*** = P<0.001; student’s t-test). (C) Results of adhesion inhibition assay. Wells of ELISA plates were coated with HeLa cells. *M*. *pneumoniae* cells were pre-incubated with different antisera and added to immobilized human cells. Detection of bound mycoplasmas was carried out with rabbit antiserum to the fraction of TX-100 insoluble proteins of *M*. *pneumoniae*. Means and standard deviations of three independent experiments with eight parallels each are shown (*** = P<0.001; student’s t-test).

To demonstrate the potential binding, HeLa cells were incubated with rPDHA-D. Bound recombinant proteins were detected with the corresponding antisera (Fig 3A). The resulting OD values were compared with the reaction of the antisera with immobilized recombinant proteins of the same concentration. For the positive control rP12 and all four rPDHA-D subunits, high binding to HeLa cells was demonstrated which exceeded the OD values obtained after incubation of recombinant proteins without cells with the corresponding antisera. Both negative controls were not able to bind to HeLa cells. The interaction between HeLa cells and the recombinant proteins was significantly reduced by pre-incubation of proteins with the corresponding antisera in comparison to guinea pig pre-immune serum (Fig 3B). To confirm the specific influence of sera on the interaction between rPDHA-D and human cells, recombinant GAPDH of *M*. *pneumoniae* was included, which was characterized as protein with strong binding to HeLa cells [22]. Using the sera to subunits of the PDH complex, an influence on the association of GAPDH to cells was missing (data not shown). However, pre-incubation of *M*. *pneumoniae* with single and pooled monospecific antisera to the PDH subunits had no measurable influence on the binding of *M*. *pneumoniae* to HeLa cells and is in accordance with the results of the antiserum to cytosolic protein enolase (Fig 3C). In contrast, pre-incubation of bacteria with antiserum to total proteins of *M*. *pneumoniae* reduced the adherence of mycoplasmas significantly.

**Fig 4:**
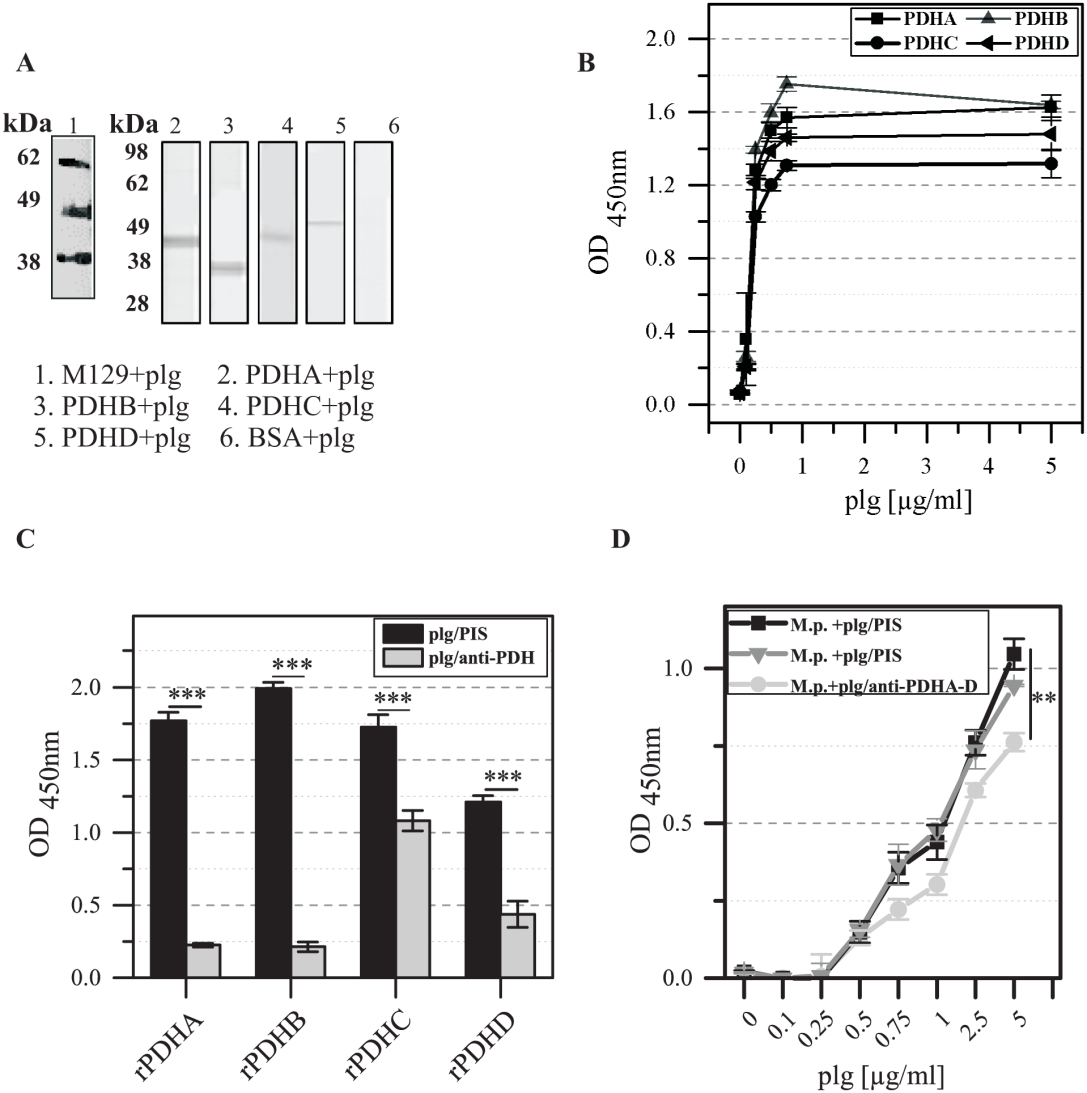
Binding of human plasminogen (plg) to recombinant proteins rPDHA-D. (A) Western blot analysis of immobilized total proteins of *M*. *pneumoniae* M129 and recombinant proteins rPDHA-D incubated with human plasminogen. Bound plasminogen was detected with rabbit anti-plasminogen and anti-rabbit IgG. Treatment of blotted BSA (66.5 kDa) served as negative control. (B) ELISA experiments to characterize the binding of different concentrations of plasminogen to immobilized rPDHA-D. Detection of plasminogen was carried out as described. Data represent means and standard deviations of eight parallels. (C) Influence of pre-incubation of rPDHA-D with the corresponding antisera on the interaction of human plasminogen with recombinant proteins. Plasminogen was pre-incubated with the sera against the subunits and added to immobilized recombinant proteins. Detection of bound plasminogen in comparison with controls using pre-immune serum (PIS) for pre-incubation was carried out by using anti-plasminogen and peroxidase-conjugated anti-rabbit IgG. Data represent means and standard deviations of eight parallels (*** = P<0.001; student’s t-test). (D) Concentration-dependent binding of plasminogen pre-incubated with the antiserum to whole proteins and with the mixture of anti-rPDHA-D to immobilized total proteins of *M*. *pneumoniae* M129 (M.p.). Plasminogen incubated with guinea pig pre-immune serum (PIS) was used as control. Means and standard deviations of eight parallels are shown (** = P<0.01; student’s t-test).

Testing the ability of separated total proteins of *M*. *pneumoniae* to bind human ECM proteins in a ligand immunoblot assay resulted in three bands after incubation with plasminogen (Fig 4A). Comparison of the molecular weight of binding partners in *M*. *pneumoniae* indicated an association of plasminogen with PDH subunits A and/or C (40.6 and 42.4 kDa) and B (35.9 kDa). Using recombinant proteins in the immunoblot, interaction of plasminogen and all four rPDH subunits is assumed (Fig 4A). This means that at least a further plasminogen-binding protein in *M*. *pneumoniae* can be expected (around 60 kDa). In addition, the interaction of recombinant protein PDHD with plasminogen is not confirmed by the investigation of whole mycoplasma cells probably caused by a low concentration and/or the particular association of the protein with components of the membrane. No signal was obtained after treatment of negative control BSA. Additional ELISA experiments showed concentration-depending binding of plasminogen to PDH subunits (Fig 4B). In comparison with guinea pig pre-immune serum, pre-incubation of human plasminogen with antisera to PDHA-D reduced the binding between plasminogen and recombinant proteins used as antigens in ELISA experiments significantly (Fig 4C). In addition, treatment with pooled antisera to PDHA-C decreased the association of *M*. *pneumoniae* cells to immobilized plasminogen concentrations > 0.5 μg/ml (Fig 4D). In contrast, pre-incubation of bacteria with the antiserum to total proteins of *M*. *pneumoniae* resulted in binding to plasminogen corresponding quantitatively to cells treated with pre-immune serum (data not shown).

### Characterization of binding of the PDH subunits to human plasminogen and of plasminogen activation

**Fig 5:**
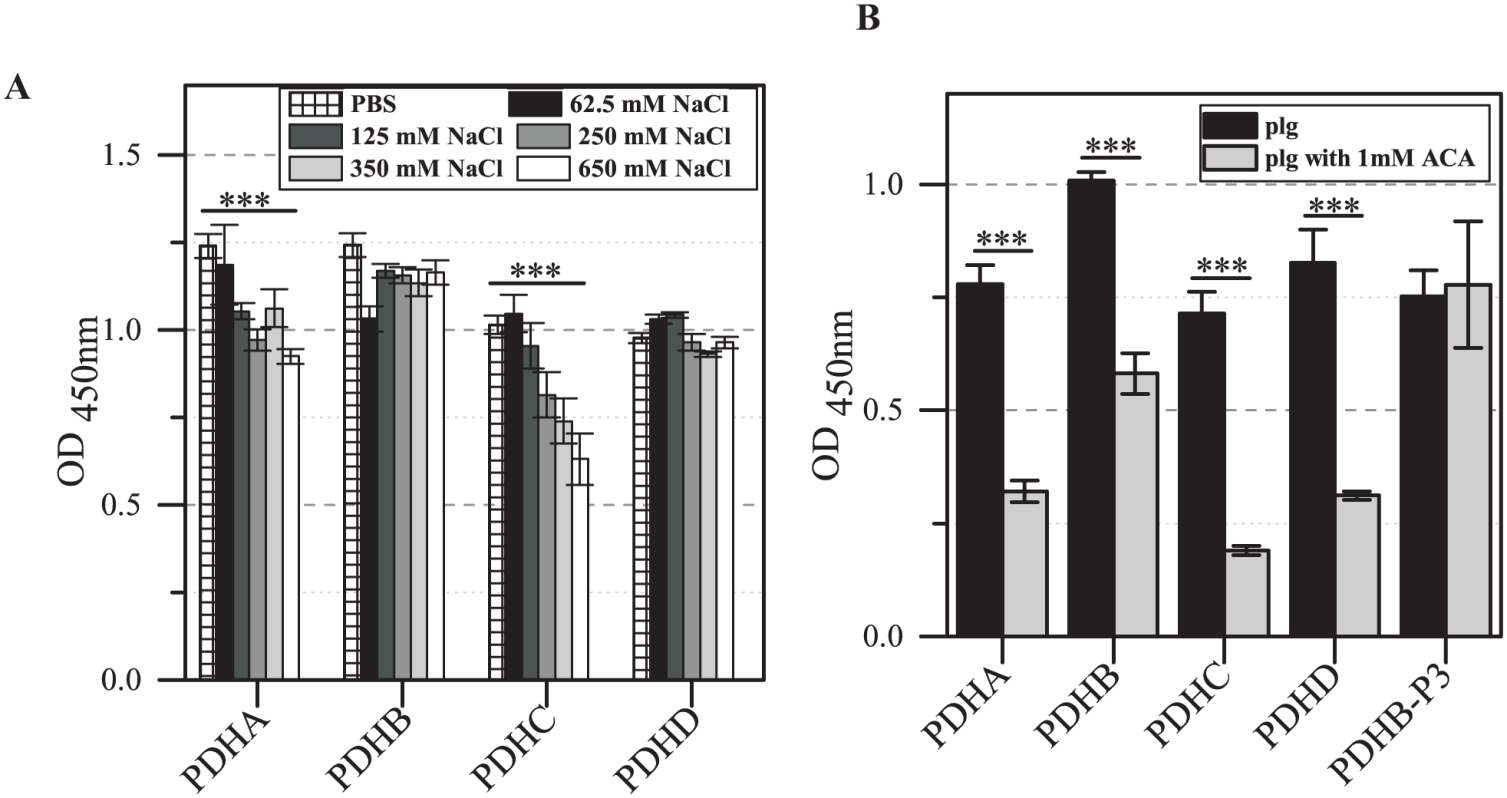
Characterization of interaction between human plasminogen and rPDHA-D. (A) The role of ionic interactions was analyzed using an ELISA assay. Recombinant proteins were immobilized to 96-well plates. Plasminogen was incubated with PBS and different concentrations of NaCl and added to the wells. Bound plasminogen was detected using rabbit anti-plasminogen. Data represent means and standard deviations of eight parallels (*** = P<0.001; student’s t-test). (B) To investigate the role of lysine, wells coated with rPDHA-D were incubated with human plasminogen in the presence of ε-aminocaproic acid (ACA). Peptide FPAMFQIFTHAA (PDHB-P3, [21]) served as negative control. Bound plasminogen was detected as described. Data represent means and standard deviations of eight parallels (** = P<0.01, *** = P<0.001; student’s t-test).

To characterize the binding of PDH subunits to plasminogen and the possible role in pathogenesis, the influence of ionic interactions, lysine residues and the activation of human plasminogen to plasmin were analyzed. Because of the high number of charged amino acids in the PDH subunits, binding assays in the presence of different concentrations of NaCl were carried out to analyze the role of ionic interactions in the association of human plasminogen with recombinant proteins PDHA-D. These experiments showed absence of an effect of NaCl regarding the binding of subunits B and D (Fig 5A). Comparable assays using heparin in increasing concentrations led to similar results (data not shown). In contrast, the presence of various concentrations of NaCl and heparin reduces binding between human plasminogen and rPDHA and C significantly. Many bacterial proteins may contain lysine residues (subunits of PDH complex: 8 to 10%) and kringle domains of plasminogen are able to bind such residues [31]. In an ELISA experiment, the addition of the lysine analog ε-aminocaproic acid reduces (between 27% and 48%) the plasminogen binding to all PDH subunits significantly (Fig 5B), indicating the important role of lysine in these interactions. The interaction of peptide 3 (^91^FPAMFQIFTHAA^102^) derived from PDHB of *M*. *pneumoniae* was not influenced by ε-aminocaproic acid.

**Fig 6:**
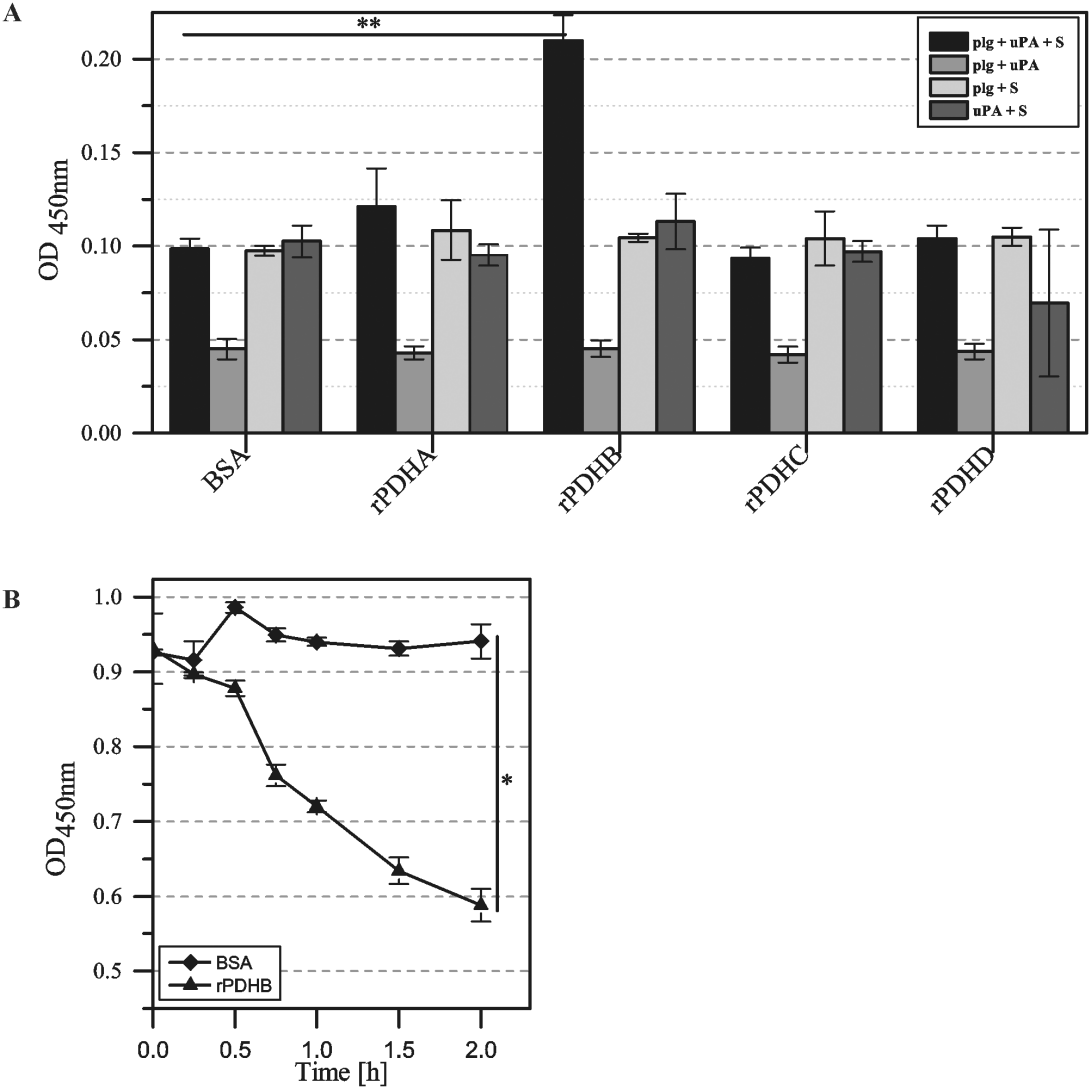
Activation of human plasminogen in the presence of rPDHA-C and degradation of fibrinogen. (A) Plasminogen bound to rPDHB is converted into plasmin. Wells of ELISA plates were coated with rPDHA-D and incubated with plasminogen. Urokinase (uPA) and plasmin-specific substrate (D-Val-Leu-Lys-*p*-nitroanilide dihydrochloride; S) were added and incubated overnight. The activity of plasmin was detected by measuring the absorbance at 405 nm. BSA acted as control. Means and standard deviations of eight parallels (** = P<0.01; student’s t-test). (B) Human fibrinogen is degraded by rPDHB. Recombinant protein rPDHB and BSA as control were immobilized in wells of ELISA plates and incubated with plasminogen. After washing, uPa and fibrinogen were added. Samples were taken at different time points up to 2 hours. Degradation products were determined using goat anti-fibrinogen and polyclonal peroxidase-conjugated anti-goat IgG. Data represent means and standard deviations of eight parallels (* = P<0.05; student’s t-test).

In the presence of an adequate activator, human plasminogen can be activated to plasmin, thus influencing the coagulation cascade. To determine the potential of rPDHA to D to activate plasminogen, recombinant proteins were consecutively incubated with human plasminogen, uPa and a plasmin-specific chromogenic substrate. Only subunit B of the PDH complex serves as activator (Fig 6A). As expected, the addition of ε-aminocaproic acid also reduced plasminogen activation (data not shown). To determine if the plasmin formation by the activator rPDHB is able to degrade the natural substrate fibrinogen, recombinant protein was immobilized to 96-well plates and incubated with plasminogen, uPA and fibrinogen. In comparison to BSA as control protein without the ability to activate plasminogen, degradation of fibrinogen by the plasminogen-rPDHB complex was confirmed (Fig 6B).

## Discussion

Surface localization is a precondition for proteins of pathogenic micro-organisms to perform important steps in host colonization such as adhesion, dissemination in tissues or immune modulation. In the present study we showed that different glycolytic enzymes of the PDH complex of a common pathogen of human respiratory tract infections are surface displayed and interact with host factors. Interaction between different proteins of bacteria, fungi and parasites and various ECM proteins is described in a multitude of studies [12]. Nevertheless, proteins with moonlighting function are of special interest in species of the class *Mollicutes* with strongly reduced genomes and therefore only a small repertoire of factors allowing their survival and proliferation in their hosts. In this context, the dual role of proteins with functions in metabolism and virulence is a way to compensate limited genetic resources. Interactions of glycolytic enzymes with host factors have been reported for *Mycoplasma gallisepticum* [15], *Mycoplasma suis* [16, 17], *Mycoplasma bovis* [18], *Mycoplasma fermentans* [19], and *M*. *pneumoniae*, respectively. In the latter, an association of the surface-localized PDHB with human fibronectin [20] as well as with plasminogen [21] was described. In addition, GAPDH of *M*. *pneumoniae* was characterized as a binding partner of human fibrinogen [22]. The findings of our study extended the spectrum of surface-localized glycolytic enzymes not only in mycoplasma species. To our knowledge, PDH subunits are not among glycolytic enzymes frequently described on the surface of bacterial species, such as enolase or GAPDH. However, PDHA-C were also detected among the *M*. *bovis* proteins inducing antibodies in naturally infected animals [32]. Recently, the E1 beta-subunit of PDH (PDHB) was found surface-expressed in *Lactobacillus plantarum* and also binds human fibronectin [33]. Summarizing the available data, a network of associations between glycolytic enzymes of *M*. *pneumoniae* and host factors can be assumed. This includes the fact that the same glycolytic enzyme (PDHB) on the cell surface is able to interact with different host proteins and also that different proteins of the bacterium bind to human plasminogen.

The results of our study confirmed that the particular PDH subunits of *M*. *pneumoniae* show different characteristics. All of them are transported from the cytosol to the membrane but only PDHA-C are surface-located. As typical for surface-exposed glycolytic enzymes, predicted signal sequences or membrane spanning motifs are also missing in the four subunits of the PDH complex of *M*. *pneumoniae*. In addition, the results of immunofluorescence experiments confirmed that the subunits of the PDH complex of *M*. *pneumoniae* were not transported to the surface of transformed *E*. *coli* BL21(DE3) cells during expression (data not shown). This indicated an unknown species-specific transport mechanism of PDH subunits to the cell surface. All proteins of the PDH complex are phosphorylated like many other enzymes in glycolysis [34] encouraging the regulation of activity and localization. Interestingly, data demonstrate that PDH subunits have limited surface accessibility. Treatment of intact *M*. *pneumoniae* cells with trypsin resulted in digestion products as well as the detection of undigested proteins PDHA-C. It can be hypothesized that glycolytic enzymes are integrated into the cell membrane of mycoplasmas exposing limited regions for interacting with host factors. This is supported by the findings of a recent study confirming a short 12 amino acid part of PDHB that is involved in plasminogen binding [21]. Probably because of the special tip structure with defined adhesins, pre-incubation of *M*. *pneumoniae* cells with the corresponding antisera to PDH subunits had no influence on the adherence of bacteria to HeLa cells displaying high concentrations of plasminogen, fibrinogen and fibronectin on their surface. The data are in accordance with results of previous studies indicating that the surface-displayed glycolytic enzymes do not take part in the primary adhesion process [21, 22]. Thus, the surface exposure of the proteins might have an impact in later phases of infection and colonization.

Plasminogen was characterized as the binding partner of various microbial proteins including glycolytic enzymes in phylogenetically different micro-organisms, suggesting a general role in the interaction with the host [35–37]. The occurrence of different glycolytic enzymes (PDHA-D or enolase [21]) with plasminogen-binding properties in *M*. *pneumoniae* emphasize the importance of this interaction. Human plasminogen is a 92 kDa serum protein and is the inactive form of the proteolytic active plasmin, which plays a key role in dissolving fibrin clots and degrades other coagulation proteins in the process of fibrinolysis [38, 39]. Here, it was shown that the four subunits of the PDH complex are able to bind to human plasminogen. Moreover, in the presence of the activator urokinase, subunit rPDHB of *M*. *pneumoniae* activates plasminogen to plasmin and the converted plasmin degrades natural substrates like fibrinogen [36]. It was hypothesized that the formation and function of plasmin will promote the dissemination of microbes across host tissue barriers and/or will optimize supplying them with nutrients. Binding of plasminogen is based on five kringle domains and these loop structures are stabilized by disulfide bonds [31]. The conformational changes after binding of lysine residues activates plasminogen to plasmin, which is found for many proteins containing lysine that serve as plasminogen binding partners in different organisms [40, 41]. The interaction between recombinant PDH subunits of *M*. *pneumoniae* and human plasminogen was characterized by addition of anions. An effect was missing in the case of PDHA, PDHB and PDHD, supporting the presumption that non-charged amino acids and conformations of the proteins rPDHA, rPDHB and rPDHD are important for interaction. In contrast, the addition of anions influenced the binding of PDHC to human plasminogen. Additionally, PDH subunit A and C has no C-terminal lysine. This confirmed the study of Crowe *et al*. [42] demonstrating that proteins without C-terminal lysine are able to bind plasminogen. Further, lysine residues bound by kringle domains mediate association with fibrinogen and other ECM proteins [43]. This binding characteristic was analyzed by the addition of ε-aminocaproic acid to the binding mixture. The substance has a high affinity to the kringle 1, kringle 4 and kringle 5 domains, but shows weak association to kringle 2 and no affinity to the kringle 3 domain [44, 45]. The presence of ε-aminocaproic acid during binding of recombinant proteins PDH to plasminogen reduced the concentration of bound plasminogen but confirmed as well that several kringle domains play a role in the interaction of the PDH subunits with human plasminogen. The broad reactivity of the kringle domains of plasminogen might explain how different glycolytic enzymes in a bacterium with limited protein resources are able to interact with human plasminogen. In contrast to other species that interact with plasminogen, e.g. *Leptospira* spp. [46], humans are the only host of *M*. *pneumoniae* in which the respiratory mucosa is infected exclusively. Thus, the data confirmed the occurrence of different microbial plasminogen binding partners despite a limited spectrum of receptors and binding sites in the naturally colonized host organisms.

## Conclusions

This is the first investigation of the localization of the subunits of a microbial PDH complex. The PDH subunits of the clinically relevant species *M*. *pneumoniae* can be found in the cytosol and in the fraction of membrane-associated proteins. In contrast to PDHA-C, PDHD does not appear to be surface-located. The derived recombinant proteins PDHA-D are able to bind specifically to human plasminogen. The results support the concept of moonlighting proteins with a dual role in metabolism and in virulence manifesting here as interaction with human plasminogen. The confirmation of a cluster of surface-displayed enzymes of a closely related part of glycolysis is a new aspect not only in the biology of micro-organisms with minimal genome. The results are of importance for the understanding of the human respiratory epithelium colonization process by *M*. *pneumoniae*.
